# Transverse myelitis masquerading as cauda equina syndrome, stroke and cervical myelopathy

**DOI:** 10.37796/2211-8039.1005

**Published:** 2020-03-28

**Authors:** Sally Shin Jie Chan, Arun-Kumar Kaliya-Perumal, Quan You Yeo, Jacob Yoong Leong Oh

**Affiliations:** Division of Spine, Department of Orthopaedic Surgery, Tan Tock Seng Hospital, Singapore

**Keywords:** compressive myelopathy, corticosteroids, neurological disorder, spinal cord compression, transverse myelitis

## Abstract

Transverse myelitis is an uncommon but well-defined neurological syndrome. However, a high index of suspicion is needed to diagnose this condition, especially when it occurs in concomitance with preexisting spinal canal stenosis. We report our patient, a 48 year old male, who initially presented to our spine clinic with acute onset unilateral lower limb weakness associated with urinary retention, which was suspected to be cauda equina syndrome due to a prolapsed intervertebral disc. However, initial magnetic resonance (MR) imaging showed only mild spinal canal stenosis from L2-L5 and C3– C6 levels; thus, the possibility of cauda equina syndrome was ruled out. A few days later, patient developed ipsilateral upper limb weakness giving an impression of hemiparesis due to stroke. However, imaging of brain returned normal. There was still a dilemma whether symptoms could be due to cervical myelopathy as there was mild cervical cord compression with early myelomalacia changes, but the findings were subtle to come to a definite conclusion. Subsequently, patient desaturated and required ventilatory support. Repeat MR imaging of the cervical spine revealed T2 hyperintensities spanning multiple levels in the cervical cord which highlighted the possibility of transverse myelitis and the diagnosis was clinched after a CSF analysis. Despite the debilitating effects, patient responded well to corticosteroid therapy and gradually recovered. This case is reported to highlight the diagnostic dilemma and the rapid progression of transverse myelitis that demands timely medical intervention to avoid permanent disabilities.

## 1. Introduction

Transverse myelitis is an uncommon condition [1]. Its annual incidence in the United States is said to be around 4.6 per million per year [2]. The incidence rate is said to be higher among those between 10 and 19 years of age, and those between 30 and 39 years of age [2]. It tends to occur irrespective of gender, race or familial predilection [2]. While it is a well-defined neurologic syndrome of the spinal cord, its etiology remains poorly defined due to the vast number of possibilities which have been proposed [3,4]. The condition is said to develop over a few hours to a few days, often termed as acute transverse myelitis; however, sometimes, it can also progress over several days. In most cases, the disease reaches its maximal clinical severity within 10 days of onset [5]. Generally, both sides of the body below the level of spinal cord lesion are affected, characterized by clear sensory demarcation [6]. However, the possibility of atypical presentations cannot be ruled out. We report a middle-aged man who was initially encountered with acute cauda equina syndrome like presentation but was later diagnosed to have transverse myelitis. In this report, we highlight the diagnostic dilemma and the rapid progression of transverse myelitis that demands timely intervention to avoid permanent neurological problems.

## 2. Case report

Our patient, a 48-year old male, had sudden onset of left lower limb weakness and numbness associated with urinary retention. There was no history of trauma or back pain. He did not present with upper limb symptoms. Except that he was a hepatitis B carrier, he did not have any preexisting medical illness. Neurological examination revealed isolated left lower limb hyper reflexia and L1 – S1 motor weakness with a power of 3/5 as per MRC grading, associated with diminished sensation over all dermatomes. There was no saddle anesthesia, rectal examination was normal, and resting anal tone was good. However, his bladder was tense and palpable which required an indwelling catheter to drain out over 1 L of residual urine. Despite his weakness, he could ambulate with use of a walking stick.

**Fig. 1:**
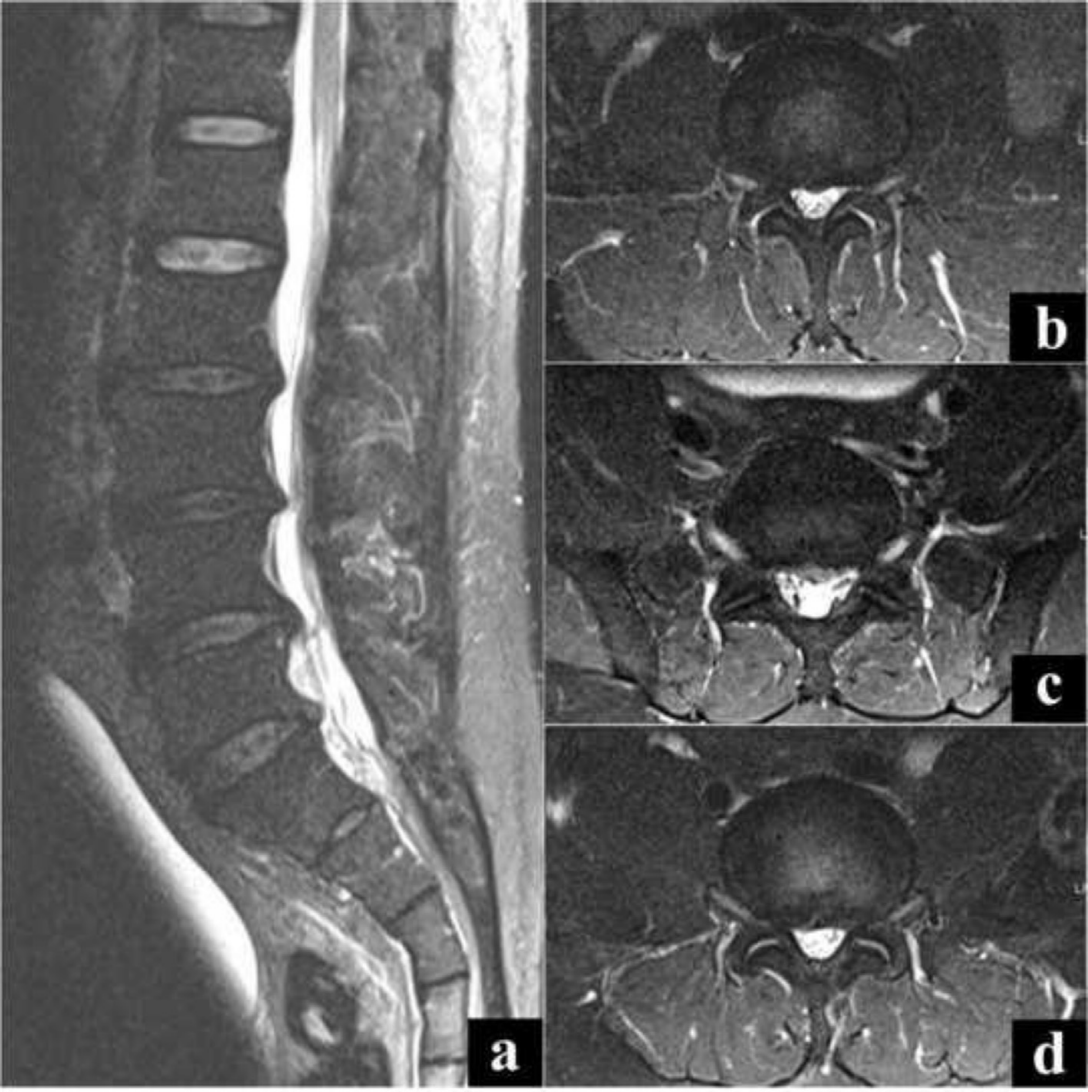
MR image showing multilevel degeneration involving L2-S1. a) Sagittal cut image, b) Axial cut at L2-L3 level, c) Axial cut at L3-L4 level, d) Axial cut at L4-L5 level.

**Fig. 2:**
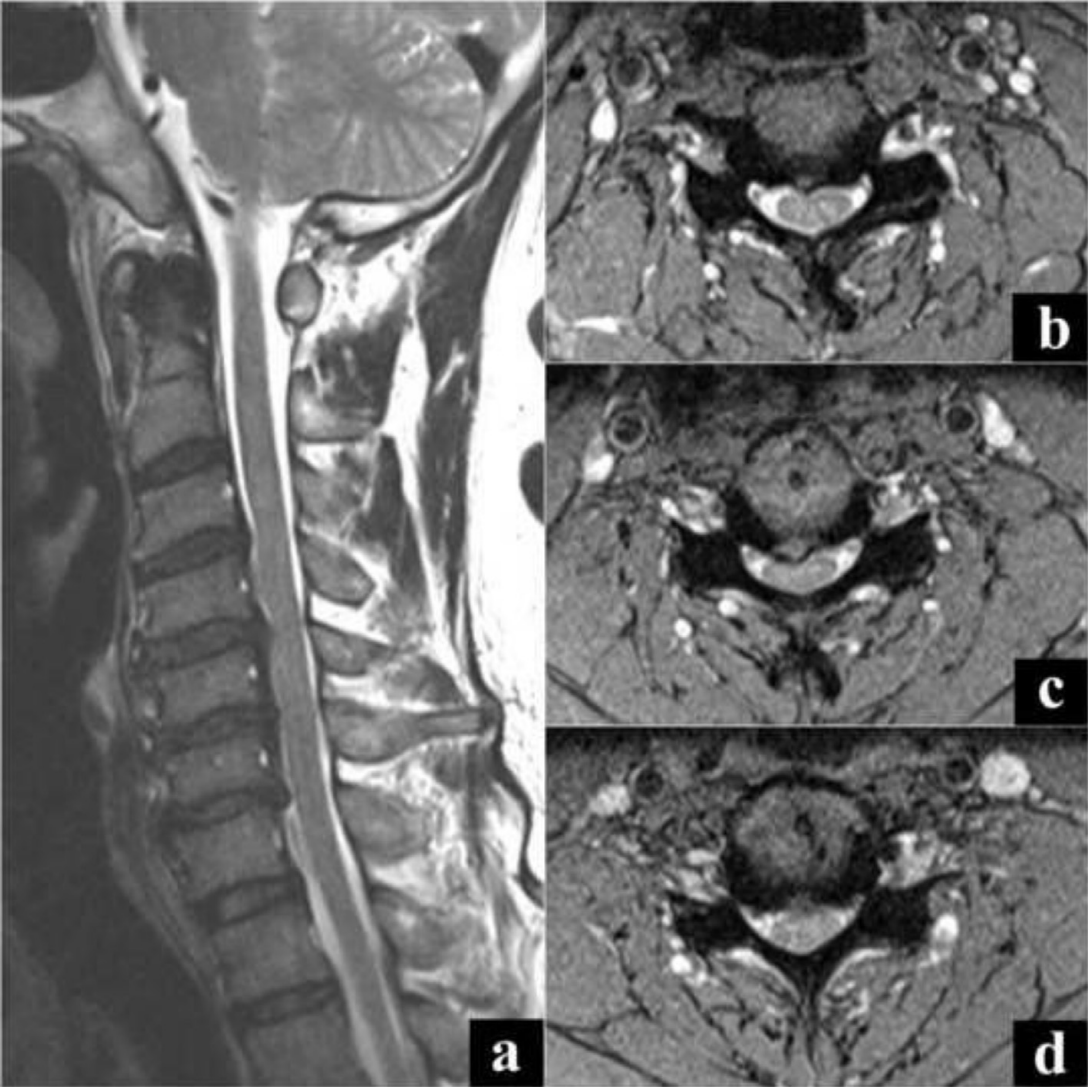
MR image showing mild spinal canal stenosis at C3–C6 levels. a) Sagittal cut image, b) Axial cut at C3–C4 level, c) Axial cut at C4–C5 level, d) Axial cut at C5–C6 level.

In view of the acute onset of lower limb symptoms associated with retention of urine, a whole spine screening MR imaging scan was ordered to look for a prolapsed intervertebral disc (PID) causing the cauda equina syndrome like presentation. However, MR imaging revealed only mild spinal canal stenosis from L2-L5 and C3–C6 levels without any cord signal changes, unlikely to cause the debilitating effects (Figs. 1 and 2). Given the clinico-radiological presentation, a definite diagnosis was not made. The patient declined further investigations and left the hospital against medical advice.

**Fig. 3:**
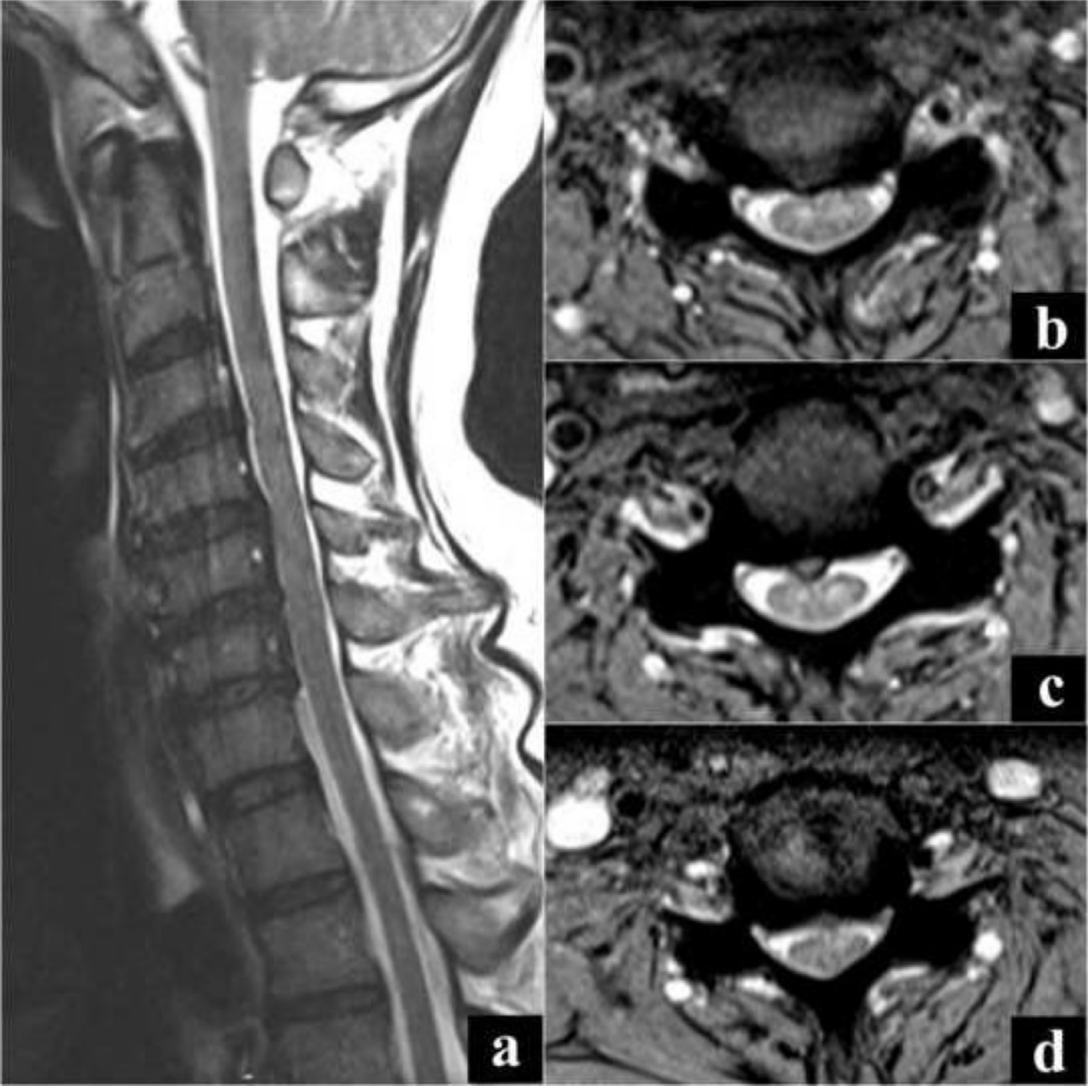
Second MR image showing mild cord signal changes on top of the C3–C6 canal stenosis seen earlier, suggestive of early myelomalacia. a) Sagittal cut image, b) Axial cut at C3–C4 level, c) Axial cut at C4–C5 level, d) Axial cut at C5–C6 level.

The following day, he returned presenting with new onset left upper limb C5 – T1 weakness with a power of 2/5 as per MRC grading and hypo-reflexia, associated with worsening neck pain. There was no neurological compromise noted in his contralateral side and Hoffman's sign was negative. A possibility of stroke was considered, and MR and CT imaging of brain was done. However, there was no evidence of infract or intracranial hemorrhage. Routine blood investigations were unremarkable. MR imaging of the whole spine was repeated which showed mild cord signal changes on top of the C3–C6 canal stenosis seen earlier, suggestive of early myelomalacia (Fig. 3). An impression of cervical myelopathy was entertained, but the presentation seemed very atypical for it.

Subsequently, five days after initial presentation, patient became acutely desaturated and increasingly drowsy, requiring emergency intubation to improve oxygen saturation. Ultrasound study revealed diaphragmatic palsy. On further clinical examination, left horner's syndrome was noted. There was completely no power in both left upper and lower limbs. In addition, there was new onset right upper limb weakness with a power of 3/5 and right lower limb weakness with a power of 2/5 as per MRC grading. Both upper limbs were hyporeflexic and both lower limbs were hyper-reflexic.

**Fig. 4:**
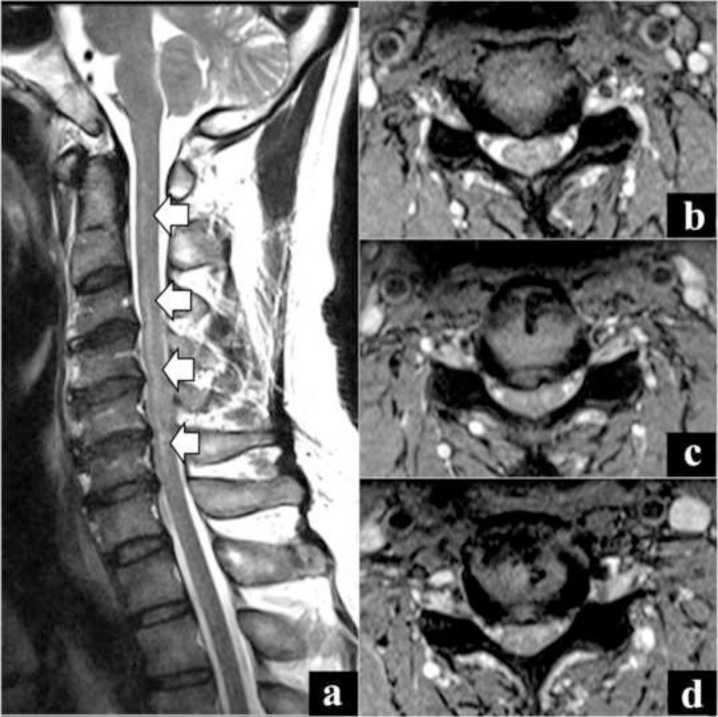
Third MR image demonstrating significant cord signal changes (T2 hyperintensities) from C2 to C7 levels. a) Sagittal cut image, b) Axial cut at C3–C4 level, c) Axial cut at C4–C5 level, d) Axial cut at C5–C6 level.

MR imaging was once again repeated which demonstrated significant cord signal changes (T2 hyperintensities) from C2 to C7 levels, without expansion or enhancement (Fig. 4). The changes were highly prominent compared to the MR imaging done previously. A diagnosis of myelitis was explored and a lumbar puncture for CSF analysis was done. CSF analysis showed the levels of glucose (5.2 mmol/L; ref. range: 2.2–4.2), protein (0.5 g/L; ref. range: 0.1–0.5) and albumin (373.0 mg/ L; ref. range: 177.0–251.0) to be high, and chloride to be normal (122 mmol/L; ref range: 120–130). Serum albumin level was slightly low (34 g/L; ref range: 35–50). The albumin CSF: Serum ratio was markedly elevated (11.0×10^−3^; ref. range: 0–8.0). NMO IgG antibody and anti-phospholipid antibodies were negative. A diagnosis of transverse myelitis was made, and the patient was started on IV Methylprednisolone 1 gm for five days followed by oral prednisolone 50 mg for 4 weeks. As he was a hepatitis B carrier, he was also started on prophylactic Entacavir in view of his high steroid dosage. Despite the debilitating effects, patient responded well to corticosteroid therapy and ventilatory support was weaned off in a week. Patient gradually regained function in 6 weeks. He was followed up every month for the first 3 months, and once every 3 months thereafter, for a period of one year. There were no signs for residual deficits and his entire follow-up period was uneventful.

## 3. Discussion

Transverse myelitis typically presents with pyramidal, sensory and/or autonomic dysfunction of varying degrees [7]. Sensory symptoms usually present as ascending paresthesia starting from the feet. Motor symptoms are generally weakness of lower limb flexors and upper limb extensors [8]. Autonomic involvement can frequently cause bowel and bladder dysfunction, temperature dysregulation, or even bouts of hypertension [8]. MR imaging lesions are typically high signal intensities in the cord, seen in T2-weighted images, demonstrating gadolinium enhancement [6]. When radiological lesions extend over three vertebral segments, then it is termed as longitudinally extensive transverse myelitis (LETM), which is often associated with a connective tissue disorder and/or neuromyelitis optica (NMO) spectrum disorder [9,10]. However, there could be other causes for LETM such as autoimmune/inflammatory diseases (multiple sclerosis, sarcoidosis or Sjögren syndrome), infection, malignancy or metabolic disturbances [10,11].

The diagnostic criteria of acute transverse myelitis as defined by the Transverse Myelitis Consortium Working Group includes: a) development of bilateral neurological signs or symptoms attributable to the spinal cord with a clearly defined sensory level and a progressive worsening to maximum between 4 h and 21 days after onset, b) absence of brain symptoms and brain MR imaging abnormalities, c) exclusion of cord compression by MR imaging and exclusion of any other etiology and d) inflammation in the spinal cord demonstrated either by cerebrospinal fluid (CSF) pleocytosis or elevated immunoglobulin G (IgG) index or MR imaging with gadolinium enhancement, at onset or within 7 days [8]. However, the presentation may vary in some cases where a high index of suspicion is needed to diagnose this condition [12,13].

While the diagnostic criteria implicate that it is important to exclude cord compression by MR imaging, this does not rule out the possibility that transverse myelitis can occur in a person with preexisting cord compression. Literature shows that there have been previous cases of patients with compressive myelopathy who were originally referred for suspected transverse myelitis [13,14]. For this reason, MR imaging can well differentiate compressive myelopathy from transverse myelitis, as the later shows gadolinium enhancement over a longer segment of the cord, while in compressive myelopathy, enhancement is limited to the region of maximal cord compression.

In our case, given the atypical presentation, the diagnosis was difficult and inconsistent, and varied at different time points over the course of the disease. Over a short span of a week, patient's condition drastically declined to the point he required ventilatory support. This signifies the rapid progression and debilitating effects of transverse myelitis. Therefore, it is essential to consider transverse myelitis as a differential diagnosis and do a CSF analysis as early as possible when encountering patients with progressive neurological deterioration without radiological evidence of a correlating lesion.

Early medical intervention with high-dose intravenous methylprednisolone is generally advised [2,15–17]. However, despite corticosteroid administration, some cases do not respond to treatment. In such refractory patients, plasma exchange is recommended [17]. The goal is to remove any humoral factors that may be contributing to the underlying pathological process and is highly effective if initiated within the first two weeks after onset of symptoms [18]. If response is still suboptimal, some authors suggest the use of IV immunoglobulin, rituximab or cyclophosphamide; however, their effectiveness remain unvalidated by larger studies [19]. Even though early improvement and response to therapy predict better outcomes, it should be remembered that approximately one third of patients remain severely disabled and some remain with moderate degree of residual disability [1,20].

## 4. Conclusion

Transverse myelitis can masquerade as other neurological and surgical pathologies. A high index of suspicion is required so that timely diagnosis and treatment can be administered to prevent its potentially life-threatening sequelae. Early medical management, which is directed to overcome the underlying inflammatory process with high-dose intravenous corticosteroid therapy, usually leads to rapid clinical improvement. However, cases refractory to corticosteroid therapy have been reported in the literature.
